# Aberrant regulation of retinoic acid signaling genes in cerebral arterio venous malformation nidus and neighboring astrocytes

**DOI:** 10.1186/s12974-021-02094-2

**Published:** 2021-03-01

**Authors:** Jaya Mary Thomas, Dhakshmi Sasankan, Sumi Surendran, Mathew Abraham, Arumugam Rajavelu, Chandrasekharan C. Kartha

**Affiliations:** 1 Cardio Vascular Diseases and Diabetes Biology, Rajiv Gandhi Centre for Biotechnology, Poojapura, Thycaud, Thiruvananthapuram, Kerala 695014 India; 2 Manipal Academy of Higher Education, Madhav Nagar, Manipal, Karnataka 576104 India; 3 Department of Neurosurgery, Sree Chitra Tirunal Institute for Medical Sciences and Technology, Thiruvananthapuram, Kerala 695011 India; 4 Pathogen Biology, Rajiv Gandhi Centre for Biotechnology, Poojapura, Thycaud, Thiruvananthapuram, Kerala 695014 India; 5 Society for Continuing Medical Education and Research, Kerala Institute of Medical Sciences, Thiruvananthapuram, Kerala 695029 India

**Keywords:** Vascular system, Retinoic acid, Astrocytes, ALDH1A2, Gene expression

## Abstract

**Background:**

Cerebral arterio venous malformations (AVM) are a major causal factor for intracranial hemorrhage, which result in permanent disability or death. The molecular mechanisms of AVM are complex, and their pathogenesis remains an enigma. Current research on cerebral AVM is focused on characterizing the molecular features of AVM nidus to elucidate the aberrant signaling pathways. The initial stimuli that lead to the development of AVM nidus structures between a dilated artery and a vein are however not known.

**Methods:**

In order to understand the molecular basis of development of cerebral AVM, we used in-depth RNA sequencing with the total RNA isolated from cerebral AVM nidus. Immunoblot and qRT-PCR assays were used to study the differential gene expression in AVM nidus, and immunofluorescence staining was used to study the expression pattern of aberrant proteins in AVM nidus and control tissues. Immunohistochemistry was used to study the expression pattern of aberrant proteins in AVM nidus and control tissues.

**Results:**

The transcriptome study has identified 38 differentially expressed genes in cerebral AVM nidus, of which 35 genes were upregulated and 3 genes were downregulated. A final modular analysis identified an upregulation of ALDH1A2, a key rate-limiting enzyme of retinoic acid signaling pathway. Further analysis revealed that CYR61, a regulator of angiogenesis, and the target gene for retinoic acid signaling is upregulated in AVM nidus. We observed that astrocytes associated with AVM nidus are abnormal with increased expression of GFAP and Vimentin. Triple immunofluorescence staining of the AVM nidus revealed that CYR61 was also overexpressed in the abnormal astrocytes associated with AVM tissue.

**Conclusion:**

Using high-throughput RNA sequencing analysis and immunostaining, we report deregulated expression of retinoic acid signaling genes in AVM nidus and its associated astrocytes and speculate that this might trigger the abnormal angiogenesis and the development of cerebral AVM in humans.

**Supplementary Information:**

The online version contains supplementary material available at 10.1186/s12974-021-02094-2.

## Introduction

Cerebral arterio venous malformations (AVMs) are an important cause for intracranial hemorrhage (ICH) and neurological morbidity in young adults [2]. The AVM nidus structures consist of abnormal blood vessels whose identity is distinct from normal vascular structures [15]. These lesions can occur in any of the four lobes of the brain, and patients with cerebral AVM present symptoms such as headache, seizures, and hemorrhage [10, 13]. The etiological factors for cerebral AVM development are not clearly understood. Earlier, we have reported that cerebral AVM nidus structures have aberrant expression of genes involved in both early and late stages of vascular development and that nidus structure of AVM co-express artery, vein, and brain capillary markers [32]. Approximately 900 genes have been identified to be associated with the pathogenesis of cerebral AVM [22]. Deregulated genes in AVM are associated with various cellular pathways that include genes regulating endocrine hormones, cell adhesion molecules, inflammatory factors, matrix metalloproteinases, and angiogenic factors [22]. It is unclear whether these genetic defects represent a primary cause for the disease or are a secondary response to disease development. Also, lack of good animal models to demonstrate the features of cerebral AVMs is impeding to decipher the pathogenic mechanisms of AVM [14].

Cerebral AVMs can develop de novo and could also be congenital in origin. The long-term notion is that AVMs are congenital in nature, and limited studies are available on the pathogenesis of this disease to genes involved in early vasculogenesis and angiogenesis. Recent reports suggest an association of cerebral AVMs with various pathological stimuli such as trauma/brain injury, viral infections, neuronal abnormal development, and migration disorders in the brain, which may occur prior to clinical manifestation of AVM [20, 21]. An investigation on the nature and functions of glial cells in the immediate vicinity of blood vessels is also possibly relevant to understand AVM pathogenesis. Any stimuli from pathological astrocytes may affect endothelial cells because of the strong anatomical and physiological interaction between astrocytes and endothelial cells. The VEGF protein is pivotal in promoting angiogenesis, and it is known that there is a high degree of VEGF expression in astrocytes in recurrent cases of AVM, when compared to non-recurrent cases of AVM [31]. There are reports of co-occurrence of AVM and astrocytoma in the brain as well [3, 24]. Interestingly, in the white matter adjoining AVMs, there is also a proliferation of oligo dendroglial cells [16–18].

Emerging high-throughput technologies open new avenues to identify the genetic defects and pathobiology of diseases at genome scale. Using microarray, the differential gene expression analysis of AVM nidus has revealed that Ephrin A1 is a candidate gene for AVM pathogenesis [27]. The transcriptome analysis of AVM nidus structures is likely to reveal deregulated molecular pathways. The RNA sequencing technology permits the study of differentially expressed genes in AVM nidus. In an attempt to identify the differentially expressed genes in cerebral AVM tissues, we have performed RNA sequencing analyses with cerebral AVM nidus structures. Since AVM nidus is heterogeneous structure, RNA sequencing was done with three independent AVM nidus samples to identify the potential changes in the pathways linked with cerebral AVM development. We identified 38 differentially expressed genes in all the three cerebral AVM samples analyzed. Among them, 35 genes were upregulated and 3 genes were downregulated. A subset of commonly differentially expressed genes belongs to retinoic acid-responsive genes. Immunohistochemical analysis revealed the presence of abnormal astrocytes in and around cerebral AVM structures. Further, we observed upregulation of CYR61, a retinoic acid-responsive gene in the abnormal astrocytes in the neighboring tissues of cerebral AVM nidus. Taken together, our study provides the results of a first in-depth transcriptome analysis of cerebral AVM nidus. We have identified that retinoic signaling pathway-associated genes are dysregulated in the vascular structures of cerebral AVM nidus.

## Material and methods

### Cerebral AVM nidus structures collection and preservation

The cerebral AVM nidus structures were collected from the patients who underwent resection of AVM at Sree Chitra Tirunal Institute for Medical Sciences and Technology (SCTIMST) between 2015 and 2017. The study was approved by the Institutional Ethical Committees of both SCTIMST and Rajiv Gandhi Centre for Biotechnology (RGCB). The control tissues consisted of vascular brain tissue from epileptic patients who had corrective surgery. Both AVM tissues and control tissues were stored immediately after resection in RNA later solution (Invitrogen, Cat No: AM7021). The transcriptome sequencing analysis was done in three independent cerebral AVM nidus and three control tissue samples, and validation studies were done using 10 AVM and 10 control tissue samples. For immunofluorescence and immunohistochemical analyses, tissues were stored in 4% paraformaldehyde (PFA).

### RNA isolation, library preparation and sequencing

RNA was isolated using TRIzol reagent (Sigma Aldrich, Cat No: 93289) as per the manufacturer’s protocol. Briefly, for up to 1 g of tissue, 500 μl of TRI reagent was added and triturated using mortar and pestle. Later, the triturate was centrifuged at 12000*g* for 10 min and the supernatant was collected in a new tube. The chloroform was added to the supernatant, and the mixture was vigorously shaken for 15 s and was allowed to stand at room temperature for 15 min. Centrifugation was again done at 12000*g* for 10 min, and the supernatant was transferred to a new tube. An equal amount of isopropanol was added to the solution and incubated at − 20 °C for 2–4 h for RNA precipitation. Later, centrifugation was done at 12000*g* for 10 min and the supernatant was decanted. Subsequently, washing of RNA was carried out in 70% ethanol and the RNA pellet was resuspended in 20 μl of 1/10 Tris EDTA buffer. The RNA concentration and quality were determined using a nanodrop spectrophotometer. A260/A280 ratio between 1.9 and 2.1 was taken to denote the quality of isolated RNA. Further, electrophoresis was carried out in formaldehyde-denatured gel and the quality of the isolated RNA was confirmed. The RNA sequencing libraries were then prepared from the isolated total RNA using Illumina True Seq mRNA standard library preparation kit; the quality of the libraries was verified using agilent tap station, and cluster generation and sequencing using NextSeq 500 were proceeded.

### Differential gene expression analysis

Differential expression analysis was performed by employing a negative binomial distribution model (DESeq v1.8.1 package http://www-huber.embl.de/users/anders/DESeq/). Genes were classified as up- and downregulated genes based on their log fold change (FC) value calculated by FC = Log2 (treated/control) formula. A heat map was constructed to show the level of gene abundance. The heat map was constructed using the log-transformed and normalized value of genes based on Euclidean distance as well as based on the average linkage method.

### Functional annotation of gene ontology analysis

Gene ontology (GO) annotations of the genes were determined by the g:Profiler program (http://biit.cs.ut.ee/gprofiler/). The g:Profiler internally uses g:GOSt which takes a list of genes and does functional profiling of them using different kinds of biological evidence. The tool performs statistical enrichment analysis to find information like Gene ontology terms and biological pathways.

### GO and KEGG enrichment analysis of differentially expressed genes (DEGs)

The functional annotations of genes were carried out against the curated KEGG genes database using KAAS (KEGG Automatic Annotation Server), (http://www.genome.jp/kegg/ko.html). Subsequently, each gene was provided with KO (KEGG Orthology) assignment, and association of the gene with KEGG metabolic pathway was determined.

### Real-time PCR analysis

Reverse transcription was done in 500 ng of total RNA using oligo(dT) primers and M-MLV reverse transcriptase as per manufacturer’s (Promega Corporation, USA) protocol. The cDNA preparations were used for the amplification of transcripts with primers specific for the genes. GAPDH was used as the endogenous control, and the expression of all genes were measured with Power SYBR Green Master Mix. The GAPDH and list of gene-specific qRT-PCR primers were used for this analysis (Supplementary Table 1). The pABI Prism 7900HT sequence detection system (Applied Biosystems, CA, USA) was used for the analysis. Thermo cycler conditions were as follows: 48 °C, 30 min; 95 °C, 10 min; 95 °C, 15 s; and 60 °C, 1 min; for 40 cycles. The ct values were generated automatically from the software SDS 2.4. Specificity of PCR products was confirmed through the detection of a single peak in the dissociation curve. mRNA fold change was calculated using the formula, 2 (ct value of target gene − ct value of control gene).

### Western blot analysis

From the tissues stored in RNA later solution, whole protein was isolated using RIPA buffer and the concentration of the protein was estimated using Bradford’s reagent. Electrophoresis of 40 μg/μl of protein was carried out in 12% SDS-PAGE gel and transferred to a nitrocellulose membrane. The nitrocellulose membrane was incubated for 1 h in 5% bovine serum albumin in Tris-buffered saline with Tween 20 to block non-specific binding sites. Primary antibody incubation was carried out for overnight. Initially, the membrane was probed with anti-rabbit ALDH1A2 antibody at a dilution of 1:1000 and later reprobed with anti-mouse GAPDH antibody in 1:5000 dilutions. The band was acquired using enhanced chemiluminescence reagent and transferred to Kodak X ray film.

### Isolation of endothelial cells from human AVM tissues and control tissues

Endothelial cells were isolated from fresh human brain AVM tissues and control tissues. Briefly, surgically excised fresh tissue samples were collected in ice cold PBS and finely minced with sterile scissors. Finely chopped tissue was transferred to 5 ml of 1 mg/ml collagenase/dispase solution and allowed to stand at 37 °C for 45 min. Later, single-cell suspension was made by aspirating the digested tissue with a 5-ml pipette. The cell suspension was passed through a 70-μl cell strainer, and the strainer was washed with medium + FBS to stop the enzymatic action. The cell suspension was then pelleted by centrifugation at 400 g for 5 min, and the supernatant was aspirated. The pellet was resuspended in 3 ml of 0.1% BSA in PBS and transferred to three 1 ml eppendoff tubes. Subsequently, the cell suspension was incubated with 22.5 μl of anti-CD31 conjugated dynabeads and tumbled at room temperature for 12 min. After tumbling, the beads were kept in the magnetic particle separator, and the supernatant was removed. Later, magnetic beads were washed five times in 0.1% BSA. Finally, RNA was isolated from the enriched cells.

### Immunohistochemical analysis

Formalin fixation of tissues was done for 2 days, and tissues were processed and embedded in paraffin. Tissue sections of 5-μm thickness were cut from paraffin blocks and were used for immunohistochemistry. The IHC supersensitive kit from Biogenex was used for immunohistochemical analysis, and the experiment was carried out as described earlier [32]. The primary antibodies were anti ALDH1A2 antibody (rabbit, 1:1000; Abcam, UK) and anti CYR61 antibody (mouse, 1:700; Abcam, UK) used for IHC analysis. Images were taken using a light microscope (Nikon Eclipse 55i microscopic system, Japan). H score analysis was done as previously described [32]

### Immunofluorescence analysis

Tissues were stored in 4% PFA overnight and subsequently suspended in increasing concentrations of sucrose in phosphate buffer saline. Tissue blocks were made by embedding them in Optimal Cutting Temperature Compound (OCT) and sectioned using a microtome. The sections were fixed in acetone and suspended in PBS. Antigen retrieval was done by incubating the tissues in citrate buffer at 97 °C for 20 s. After blocking of non specific binding sites using 3% bovine serum albumin (BSA), overnight incubation with primary antibody was carried out. For single immunofluorescence staining with ALDH1A2 antibody overnight incubation (1:100 dilutions) was done. For double and triple immunofluorescence staining, all the primary antibodies in 1:100 dilutions, anti GFAP (goat, Abcam, UK), anti Vimentin (mouse, Abcam, UK), anti ALDH1A2, and anti CYR61 were added simultaneously and incubated overnight.

Excess primary antibody was washed off using PBST, and incubation with Alexa Fluor conjugated rabbit/mouse/goat secondary antibodies (1:500, Abcam UK) was done for 1 h. The Hoechst dye was used for staining the nucleus, and mounting was done using fluorochrome; the images were collected using a fluorescent microscope (Leica, × 60 magnifications).

### Statistical analysis

Graph Pad PRISM version 6.07 was used for statistical analysis. All the results were expressed as mean ± SEM. Student’s *t* test was done to analyze difference in mRNA and protein expression levels. The *P* value of less than 0.05 was the criterion to be statistically significant.

## Results

### Transcriptome profiling of cerebral AVM nidus identifies aberrant gene expression

**Fig. 1:**
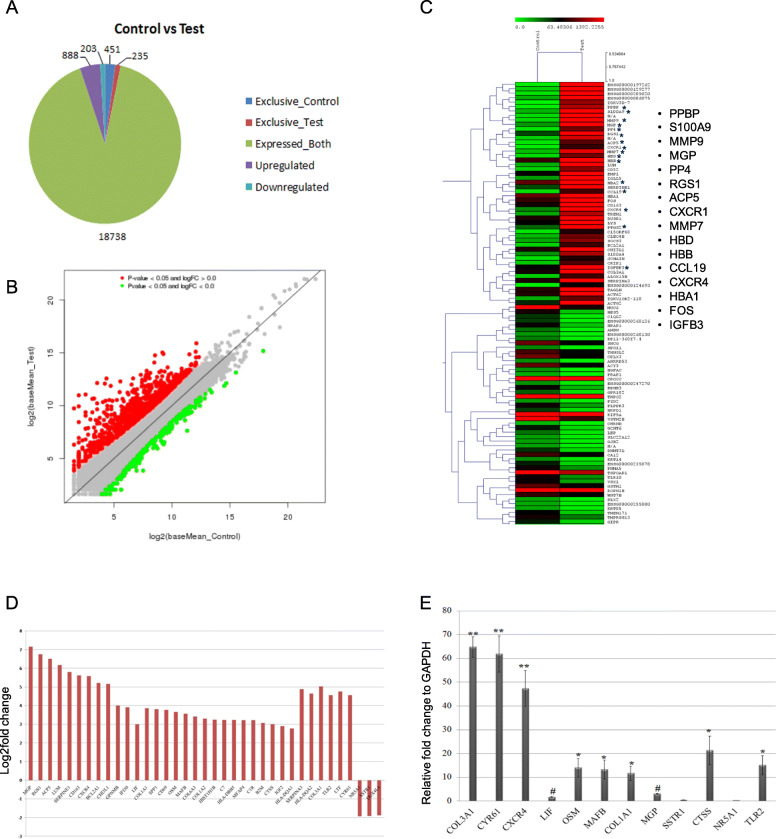
Transcriptome analysis of cerebral AVM nidus and controls tissues. **a** Pie diagram showing gene distribution of differentially expressed genes in arterio venous malformation (AVM) nidus samples compared to control sample. **b** The scatter plot representation of differentially expressed genes in cerebral AVM nidus, which allows to identify the expression levels of genes in two distinct conditions. In the scatter plot, each dot represents a gene, and thus, genes that fall above the diagonal are overexpressed and genes that fall below the diagonal are underexpressed in AVM. **c** Top 100 differentially expressed genes in the cerebral AVM tissues compared to control brain tissues are represented as heat map. The asterisk marked genes represent the genes involved in blood vessel development. The color represents the logarithmic intensity of the expressed genes. Relatively high expression values are shown in red color. **d** The bar plot represents the Log2 fold change in the expression of commonly differentially expressed genes in the cerebral AVM samples compared to control samples. A total of 38 genes were differentially expressed commonly in all the three independent AVM samples analyzed compared to control samples, among which 35 genes were upregulated and three genes viz, NR5A1, SSTR1, and DUX4L4, were downregulated. **e** Validation of differentially expressed genes common in three independent samples of cerebral AVM nidus. The following genes were validated by qRT-PCR. COL3A1, CYR61, OSM, MAFB, COL1A1, CTSS, TLR2, and CXCR4, and their expression were normalized to control samples, and internal normalization was carried out using GAPDH. Many tested genes were significantly up regulated in AVM tissue. The LIF and MGP gene expression were not significantly increased in AVM compared to control tissue. The qRT-PCR was carried out with 10 AVM nidus and 10 control samples, and the GAPDH was used as endogenous control for normalization. Individual paired *t* test was performed to calculate the *P* values (*P < 0.05 and **P < 0.01)

To identify the aberrant molecular signatures in the cerebral AVM nidus structures, we employed in-depth RNA sequencing with RNA isolated from cerebral AVM nidus. The quality of the RNA was verified by formaldehyde-denatured agarose gel (Supplementary figure 1A), and RNA was subjected to fragmentation and library preparation (Supplementary figures 1B–1C) and proceeded to sequencing. The qualities of RNA sequencing reads were very good in both controls and test samples, and 97.54% of sequencing reads for AVM nidus tissues and 91% reads in control tissues were mapped to the human reference human genome. After stringent comparisons, we identified 888 genes were upregulated and 203 genes were downregulated in cerebral AVM nidus tissues compared to control tissues (Fig. 1a, b). The top 100 differentially expressed genes were subjected for hierarchical cluster analysis using multiple experiments viewer (MeV v4.9.0), and the results are provided as a heat map representation (Fig. 1c). The gene products were further grouped into those linked to biological processes, molecular functions, and cellular components (Supplementary figures 2A–2B). The differential gene expression (DGE) analysis was performed to identify the aberrantly expressed genes, and based on enrichment analysis, DGE were classified into 41 functional pathway-related genes (Supplementary figure 3).

An absolute cutoff value of log2 fold change up to 1.5 has been taken to identify the commonly differentially expressed genes (CDEGs) in the three AVM samples when compared to control samples (Fig. 1d). The CDEGs included both upregulated and downregulated genes. We observed 38 CDEGs of which 35 genes were upregulated and 3 genes were downregulated (Table 1). Interestingly, we observed a subset of genes that are involved in retinoic acid signaling in CDGEs and another subset of genes involved in regulation of inflammation and immune responses (Table 2). Further, we validated the deregulated genes by qRT-PCR in 10 different cerebral AVM and control tissues and found consistent upregulation and downregulation as we have observed in the transcriptome analysis (Fig. 1e). Previously, we reported that cerebral AVMs are terminally undifferentiated and contains a mixture of arteries, veins, and capillaries [32], and current RNA sequencing data identifies an aberrant expression of genes in cerebral AVMs.

**Table 1:** List of commonly differentially expressed genes in AVM nidus

**Up regulated**	MGP, RGS1, ACP5, LUM, SERPINE1, CD163, CXCR4, CXCL12, BCL2A1, CHI3L1/L2, GPNMB, IFI30, LIF, COL1A1, SPP1, CD69, OSM, MAFB , COL6A3, COL1A2, HIST1H1B, C7, HLA-DRB5, MFAP4, C1R, B2M, CTSS, IGF2, HLA-DQA1, SERPINA3, HLA-DQA2, COL3A1, TLR2, LTF and CYR61
**Down regulated**	NR5A1, SSTR1, and DUX4L4

**Table 2:** Classification of commonly differentially expressed genes in the three AVM nidus samples

**Retinoic acid signaling pathway genes**	MGP, SERPINE1, COL1A1, SPP1, MAFB, SERPINA3, COL3A1, CYR61
**Genes related to inflammation and immune response**	CD163, CXCR4, CHI3L1/L2, IFI30, LIF, CD69, OSM, C7, HLADRB5, C1R, CTSS, HLA-DQA1, HLA-DQA2, TLR2, LTF

### Dysregulation of retinoic acid signaling genes in cerebral AVM nidus

**Fig. 2:**
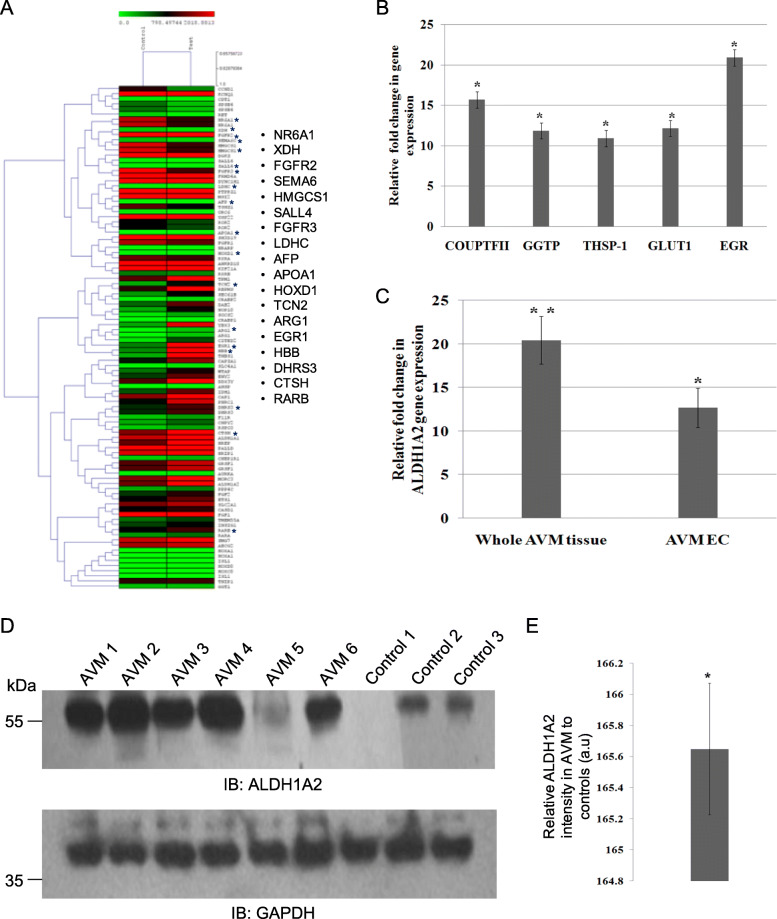
Analysis of aberrantly expressed retinoic acid-responsible genes in AVM nidus. **a** The heat map representing retinoic acid response genes from the RNA sequencing analysis that are differentially expressed in the cerebral AVM nidus. The genes that are directly or indirectly associated with vascular development are marked with an asterisk, and the list of marked genes are provided in the expanded version. **b** Validation of selected retinoic acid response genes such as COUP-TFII, GGTP, THSP-1, GLUT1, and EGR in AVM by qRT-PCR analysis and the expression of these genes are represented by fold change calculated from 10 different AVM nidus samples and 10 number of control tissues (*P < 0.05). The GAPDH was used as the endogenous control for normalization. **c** The qRT-PCR analysis for ALDH1A2 in AVM and control samples. The fold change in the gene expression of ALDH1A2 in AVM tissues was compared to that of control tissues, and the endothelial cells isolated from AVM (AVMEC) were compared to endothelial cells isolated from control tissues. Analysis was carried out with 10 AVM and 10 control tissues, and endothelial cells were isolated from 5 AVM samples and 5 control samples. The GAPDH was used as endogenous control for normalization (*P < 0.05). **d** The Western blot analysis of ALDH1A2 protein for the AVM tissues and control tissues. The bottom immunoblot represents the GAPDH for loading control, probed on the same blot used for ALDH1A2 protein. **e** The densitometry analysis of ALDH1A2 protein and the relative intensity in AVM tissue compared to control tissues. The ALDH1A2 expression is significantly increased in AVM tissues compared to control tissues (*P < 0.05). The GAPDH is the loading control. AVM (*n* = 11) and control (*n* = 7)

**Fig. 3:**
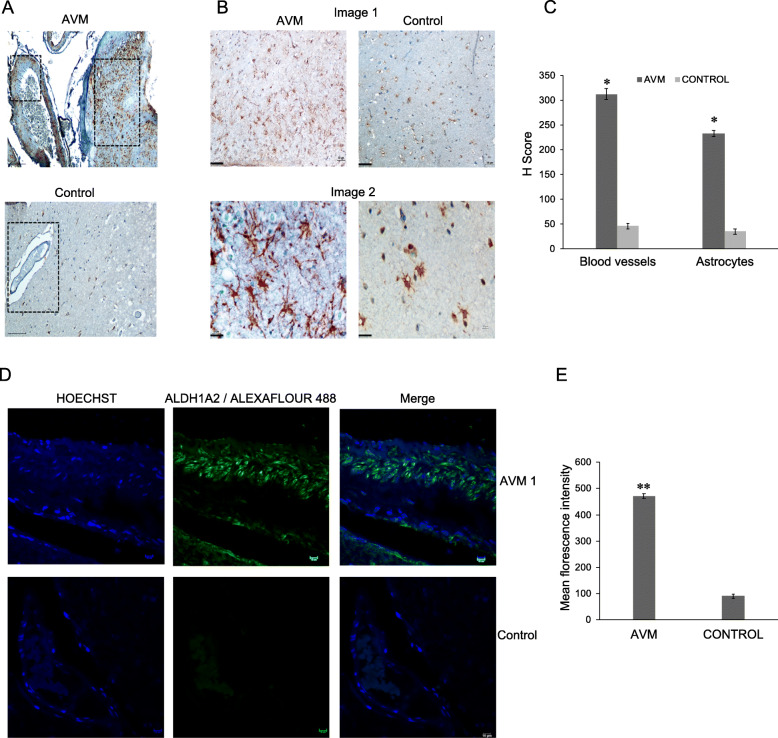
Immunostaining of ALDH1A2 in cerebral AVM nidus and control tissues. **a** Representative image of immunohistochemical analysis with ALDH1A2 antibody in cerebral AVM and control tissues shows increased expression of ALDH1A2 in AVM blood vessels. In control tissues, there is no expression of ALDH1A2 in the blood vessel. The images were collected with × 20 magnification (scale bar, 100 μm). **b** The immunohistochemical analysis of cerebral AVM and its associated astrocytes shows increased expression of ALDHA2 protein compared to astrocytes in the control tissue. Image 1 shows × 10 magnification and image 2 shows × 40 magnification (scale bar 100 μm). **c** The bar plot represents the H score analysis of ALDH1A2 protein expression. There is increased expression of this protein in AVM tissue compared to control (*P < 0.05), AVM (*n* = 10) and control (*n* = 10). **d** Immunofluorescence analysis of cerebral AVM and control tissues with ALDH1A2 shows upregulation in AVM blood vessel (green). In control vessels, there is less expression of ALDH1A2 protein. The Hoechst 33342 (blue) dye is used to counter stain nuclei of the cells. The images were collected using × 60 magnification (scale bar, 10 μm). **e** The bar plot represents the mean fluorescence intensity of ALDH1A2 in AVM and control blood vessel. There is an increased expression of ALDH1A2 in AVM blood vessel compared to control (*P < 0.05), AVM (*n* = 10) and control (*n* = 10)

To understand the molecular changes in the cerebral AVM nidus structures, we performed pathway analysis with transcriptome data sets and found a distinct pattern of differential gene expression in AVM tissue compared to control tissues (Fig. 2a). The highly deregulated genes belong to a subset of retinoic acid-responsive genes, particularly MGP, SERPINE1, COL1A1, SPP1, MAFB, SERPINA3, COL3A1, and CYR61 (Fig. 2a). We validated the expression changes using qRT-PCR analysis and found a significant upregulation of retinoic acid response genes such as COUP-TFII, GGTP, THSP-1, GLUT1, and EGR in cerebral AVM nidus (Fig. 2b). To identify the master regulator of these changes, we analyzed the expression of ALDH1A2, a key rate-limiting enzyme of retinoic acid signaling pathway by qRT-PCR analysis in 10 AVM nidus and 10 control samples. The results have identified a significant upregulation of ALDH1A2 protein in cerebral AVM nidus compared to control tissues (Fig. 2c). To validate these findings, we have isolated endothelial cells from five AVM nidus tissues and analyzed the expression of ALDH1A2 gene. The qRT-PCR analysis revealed that ALDH1A2 gene is upregulated in endothelial cells isolated from AVM tissues than normal brain capillary endothelial cells isolated from control tissues (Fig. 2c). We have studied the expression of ALDH1A2 in the protein levels of AVM nidus and control tissues and found consistent upregulation of ALDH1A2 protein in AVM nidus tissues than in control tissues (Fig. 2d, e). To substantiate further, we performed the immunohistochemical analysis for ALDH1A2 in cerebral AVM and control tissues. It is evident that there is an elevated expression of ALDH1A2 in the blood vessels (Fig. 3a, c), and surprisingly, we have found increased levels of ALDH1A2 in the surrounding astrocyte cells in AVM tissue (Fig. 3b, c). Further, we have performed an immunofluorescence analysis with ALDH1A2 antibody in the AVM and control tissues and observed that AVM-associated blood vessels had an elevated expression of ALDH1A2, whereas very less expression was observed in control tissue vessels (Fig. 3d, e). Immunoblot and immunohistochemical analyses of cerebral AVM nidus confirms the upregulation of ALDH1A2, a key rate-limiting enzyme of retinoic acid signaling pathway, which in turn controls the many angiogenic genes in the vascular system (Fig. 2a). The deregulation of ALDH1A2 in AVM nidus proposes potential changes in vasculogenesis that may lead to the development of cerebral AVMs.

### Aberrant expression of ALDH1A2 and CYR61 in abnormal astrocytes in the neighborhood of AVM nidus

**Fig. 4:**
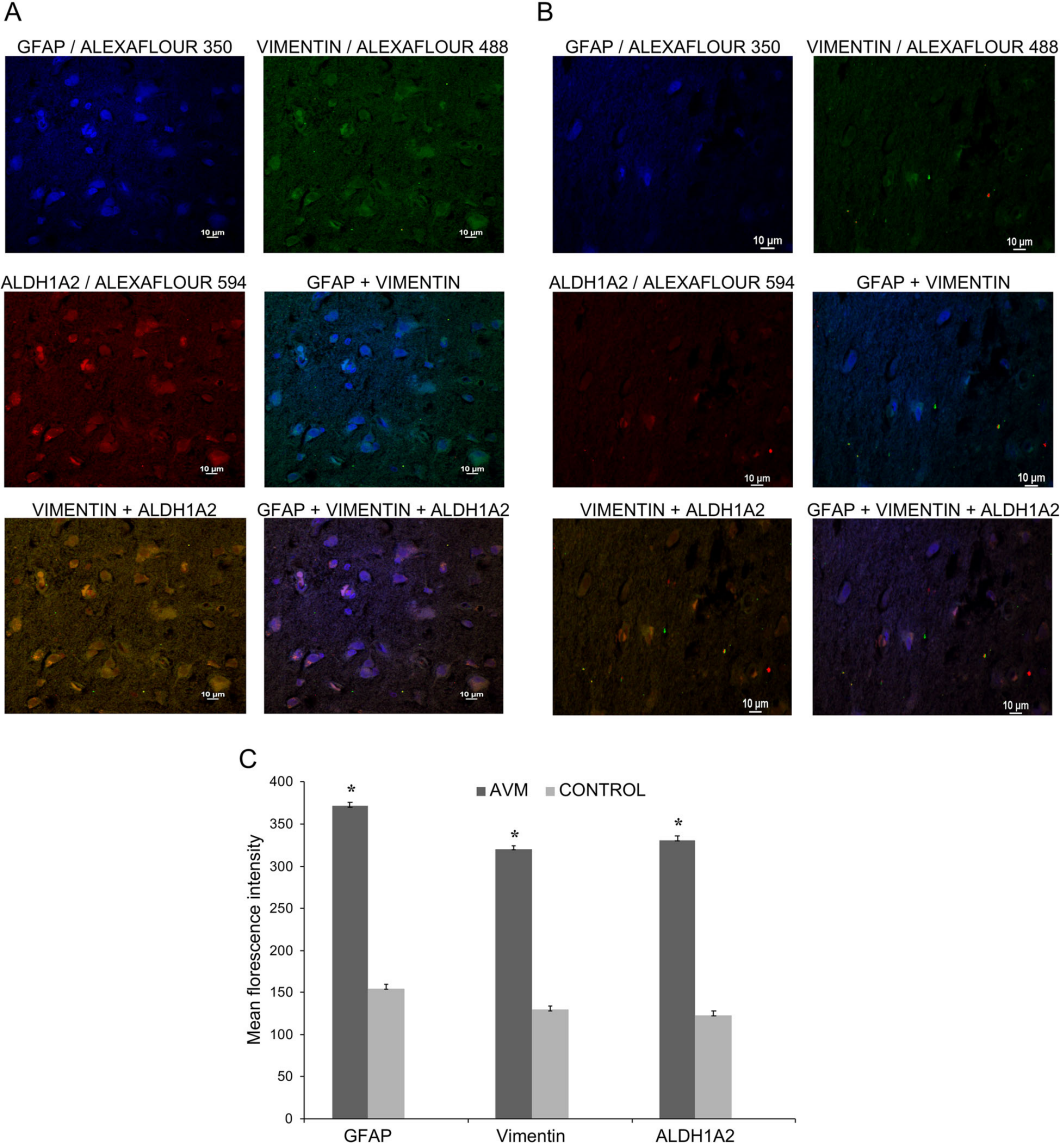
Triple immunofluorescence staining analysis for GFAP, Vimentin, and ALDH1A2 in cerebral AVM and control tissues. **a** Triple immunofluorescence staining with GFAP (blue), Vimentin (green) and ALDH1A2 (red) with cerebral AVM nidus. The immunofluorescence staining with GFAP, Vimentin, and ALDH1A2 reveals high expression of these proteins in astrocytes surrounding the AVM vessel. The GFAP + ALDH1A2 + Vimentin immunofluorescence staining demonstrates the co-localized expression of these three proteins in AVM-associated astrocytes. **b** Triple immunofluorescence staining with GFAP (blue), Vimentin (green), and ALDH1A2 (red) in control tissue, which reveals the minimal expression of ALDH1A2. The images were collected using × 60 magnification (scale bar, 10 μm). **c** The bar plot represents the mean fluorescent intensity analysis of GFAP, Vimentin and ALDH1A2 in AVM and control vessel. There is a significantly increased expression of all these proteins in AVM tissue compared to control (*P < 0.05), AVM (*n* = 10) and control (*n* = 10)

The cerebral vascular system is enriched with astrocytic cells, and the presence of abnormal astrocytes around human cerebral AVMs is reported [17, 18]. Since there was an upregulation of ALDH1A2 protein in AVM nidus, we were interested to analyze the expression of ALDH1A2 protein in abnormal astrocytes around the cerebral AVM nidus. To study this, we have performed triple immunofluorescence (IF) staining for GFAP and Vimentin, well-known marker proteins for abnormal astrocytic conditions [12]. Along with these proteins, we have performed IF staining for ALDH1A2 protein to AVM nidus and control tissues. The IF staining has clearly indicated that there is strong upregulated expression of GFAP, Vimentin, and ALDH1A2 in abnormal astrocytes associated with cerebral AVMs (Fig. 4a, c). Whereas, the triple immunofluorescence staining with all three antibodies in the control tissues has revealed minimal expression of these marker proteins in astrocytes associated with control tissues (Fig. 4b, c). The specificity of primary antibodies used in this study was verified by immunostaining of AVM tissues with secondary antibodies, and the results confirm the absence of localization-specific signals in the tissues (Supplementary figure 4). This data supports that upregulation of ALDH1A2 in cerebral AVM and its associated abnormal astrocytes; thus, we speculate that the abnormal astrocytes might be a potential stimulus for the development of cerebral AVMs in humans.

**Fig. 5:**
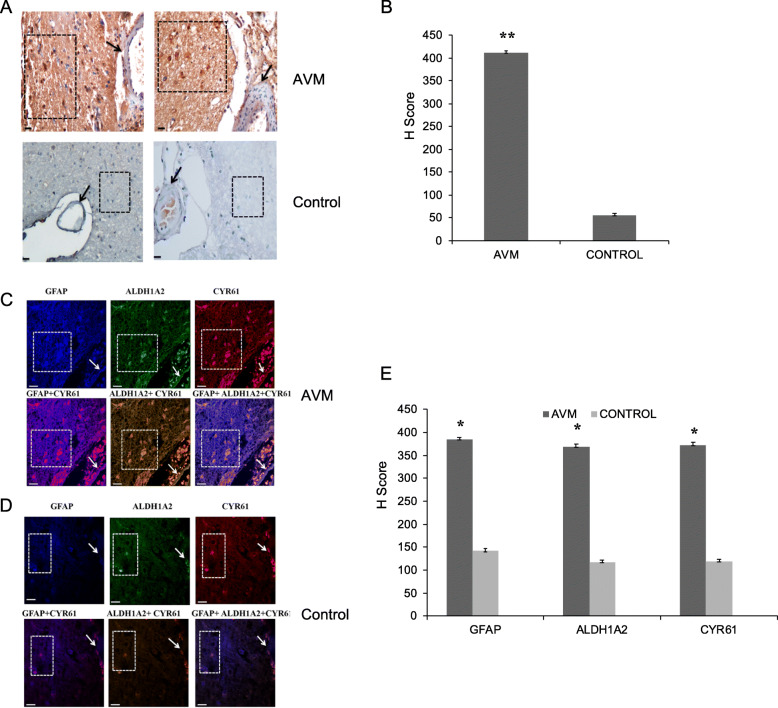
CYR61 expression analysis in cerebral AVM nidus and its neighboring astrocytes. **a** Representative images of immunohistochemistry analysis for CYR61 protein using antibody with cerebral AVM nidus and control tissues. The CYR61 is highly expressed in AVM brain parenchyma (box) and less expressed in AVM blood vessel (arrow). In control tissues, the CYR61 protein is not expressed in brain parenchyma (box) as well as in control blood vessel (arrow). The images were collected using × 20 magnification (scale bar, 100 μm). **b** The bar plot represents the H score analysis of CYR61 expression in AVM and control tissue. There is an increased expression of this protein in AVM tissues compared to control tissues (**P < 0.01), AVM (*n* = 10) and control (*n* = 10). **c** Photomicrograph representing triple immunofluorescence staining with GFAP (blue), ALDH1A2 (green), and CYR61 (red) in cerebral AVM tissues. Immunofluorescence staining with GFAP, ALDH1A2, and CYR61 reveals high expression of these proteins in astrocytes (box) surrounding the AVM vessel (arrow). The GFAP+ ALDH1A2 + CYR61 triple immunofluorescence staining demonstrates the co-localized expression of these three proteins in AVM-associated astrocytes. **d** The triple immunofluorescence staining with control tissues shows minimal expression of all the three proteins in astrocytes (box) surrounding the control blood vessel (arrow). **e** The bar plot represents the mean fluorescent intensity analysis of GFAP, ALDH1A2, and CYR61 in AVM and control vessels. There is an increased expression of these proteins in AVM compared to control tissues (*P < 0.05), AVM (*n* = 10) and control (*n* = 10)

RNA sequencing data and qRT-PCR analysis have identified increased expression of one of the retinoic acid response genes CYR61 (Fig. 2a). The CYR61 is a well-known protein that is involved in angiogenesis. Since we observed upregulation of ALDH1A2 protein in abnormal astrocytes surrounding the cerebral AVM nidus, we analyzed the expression of CYR61 protein in the astrocytes surrounding the cerebral AVMs. To study this, we performed immunohistochemical analysis with CYR61 antibody in the cerebral AVM and control tissues and observed high expression of CYR61 protein in the brain parenchyma cells surrounding the AVM vessels and reduced expression in AVM blood vessels (Fig. 5a, b). In contrast to this, we observed very low expression of CYR61 protein in blood vessels as well as brain parenchyma cells of control tissues (Fig. 5a, b). To substantiate this finding, we analyzed the expression of CYR61 in astrocytes using triple immunofluorescence staining of AVM nidus and control tissues with GFAP, ALDH1A2, and CYR61 antibodies. We observed increased expression of CYR61 in astrocytes around the AVM and strongly co-localized to GFAP, ALDH1A2. However, we observed very minimal expression of these proteins in the astrocytes of control tissues (Fig. 5c–e). Taken together, our study provides the direct evidence that abnormal astrocytes surrounding the cerebral AVMs contain increased expression of ALDH1A2, a key rate-limiting enzyme of retinoic acid signaling and its target protein CYR61. In conclusion, this study proposes that development of cerebral AVM nidus could be due to multifactorial stimuli and is possible within the vascular cells as well as from the adjacent abnormal astrocyte cells.

## Discussion

Transcriptomic analysis of nidus tissue of human cerebral AVM reveals upregulation of the retinoic acid target angiogenic gene CYR61 in the AVMs and the astrocytes in and around the cerebral AVM nidus. The upregulation of CYR61 was associated with increased expression of ALDH1A2 protein, a key rate-limiting enzyme of retinoic acid signaling pathway. This novel finding indicates the role of astrocytes in modulating angiogenesis in the brain parenchyma around AVM.

Gene expression profiling studies strongly support the identification of differentially expressed genes in the AVM and thus the discovery of potential marker genes that can be used in the diagnosis and predicting the prognosis of this disease. No targeted study has been done in human cerebral AVMs to identify the potential pathways and genes involved in the pathogenesis of the disease. We have identified 37 commonly differentially expressed genes (CDEGs) in AVM nidus tissues. These CDEGs could be classified into two groups: 1) genes related to inflammation and immune response and 2) genes regulated by retinoic acid signaling. Earlier reports have suggested the presence of inflammatory cells in AVM tissues. Although not considered as an inciting event in cerebral AVM pathogenesis, the role of inflammation in the clinical course of this disease is widely recognized. Inflammation is associated with disease progression and rupture of AVM [9, 23]. It is known that the risk for intracranial hemorrhage (ICH) in AVM is linked to polymorphisms in the inflammatory cytokine genes such as TNF-α and IL-6 [1, 25].

The role of retinoic acid signaling in regulating angiogenesis is not studied in detail. A few reports have suggested that retinoic acid has both anti-angiogenic and pro-angiogenic effects [11, 19, 26]. All-trans retinoic acid (ATRA), the key molecule in retinoic acid signaling, has an important role in modulating angiogenesis. In vitro co-culture studies with human dermal fibroblasts (NHDFs) and human umbilical vein ECs reveal that the dose dependent role of ATRA in stimulating capillary tube formation, through activation of endogenous VEGF signaling [26]. Retinoids are also known to inhibit arterial formation that results in decreased artery to vein ratio. Further, retinoids reduce the vessel length and the number of arteries [5]. These observations indicate the potential role of retinoic acid signaling in regulating normal blood vessel formation. Our study demonstrates for the first time that retinoic acid signaling pathway and their target genes are dysregulated in cerebral AVM nidus. Moreover, we observed increased expression of ALDH1A2, the rate-limiting enzyme in the retinoic acid signaling pathway and various retinoic acid response genes in the malformed vascular structure of AVM nidus (Fig. 2a).

Upregulated expression of ALDH1A2 in astrocytes in the immediate neighborhood of cerebral AVM nidus structures is an interesting finding of our study. This observation prompted us to analyze the nature of the astrocytes surrounding the AVM, and we observed that the astrocytes of the AVM nidus had high expression of Vimentin and GFAP, which suggest the abnormal nature of these astrocytes. There was an increased expression of Vimentin, GFAP, and ALDH1A2 in cerebral AVM-associated astrocytes. It is known that astrocytes of the normal adult brain have very less or undetectable levels of ALDH1A2, whereas astrocytic gliomas and in proliferative states of astrocytes contain elevated levels of ALDH1A2 [6].

Among the differentially expressed genes in AVM vascular structures, the CYR61, one of the retinoic acid target genes, was highly deregulated in the cerebral AVM-associated astrocytes and vascular cells. The CYR61 protein controls the critical regulators of the angiogenesis process. Angiogenesis is under the control of vascular endothelial growth factor (VEGF) which in turn is regulated by CYR61 [7]. The CYR61 facilitates angiogenesis by inducing the mitogenic activity of various growth factors, promoting endothelial cell migration, adhesion, etc. The CYR61 also induces the expression of αv integrin subunit and matrix metalloproteinase, other genes involved in angiogenesis [8, 35]. The CYR61 is not only involved in physiological angiogenesis but also in increased angiogenesis associated with pathological conditions such as tumors, where its increased expression promotes vascularization [4].

Another interesting observation of our study is the increased concomitant expression of GFAP, Vimentin, ALDH1A2, and CYR61 in the cerebral AVM-associated astrocytes. The CYR61 is a matricellular protein that is secreted by astrocytes during development. Expression of this protein is reduced in the adult brain. It is known that the CYR61 is upregulated during tissue remodeling, axon regeneration, and angiogenesis. Expression of CYR61 has also been found to be increased in various types of tumors such as pancreatic cancers, melanomas, mammary tumors, and various grades of primary gliomas [30]. Recently, it was reported that in AVM tissue, there is an increased expression of integrin alphavbeta1 [28, 29], whose expression is induced by CYR61 protein [33, 34]. Therefore, we speculate that the CYR61 over expression in the cerebral AVM-associated astrocytes could be an initial stimulus that activates downstream pathways leading to aberrations in differentiation of vascular structures and maturation. We conclude that CYR61 from abnormal astrocytes in pathological conditions may have a role in inducing the formation of aberrant vascular structures as seen in the AVM nidus.

## Supplementary Information

**Additional file 1:** **Supplementary Figure 1.** (A) Formaldehyde denature agarose gel depicts the quality and intactness of RNA isolated from control and test (AVM nidus) tissues. (B) The library prepared from fragmented RNA of control sample and the quality of the library was verified using Agilent tap station. (C) The library prepared from fragmented RNA of AVM nidus sample and the quality of the library was verified using Agilent tap station. **Supplementary Figure 2.** (A) WEGO plot representing the up-regulated genes in AVM tissue compared to control tissue. (B) WEGO plot representing the down-regulated gene in AVM tissue compared to control tissue. **Supplementary Figure 3.** The KEGG pathway analysis of AVM tissue sample and control tissue sample of RNA sequencing data. **Supplementary Figure 4.** The immunofluorescence analysis with the secondary antibodies as control for AVM tissues. All the three secondary antibodies used in the analysis has zero background signal. The Hoechst staining depicts the nuclear staining of the intact cells.

**Additional file 2:** **Supplementary Table 1.** List of primers used for qRT-PCR analysis in this study.

## Data Availability

The authors declare that the data generated in this study are available with in the article, and its supplementary information, or are available from the authors upon request.

## Abbreviations

AVM: Arterio venous malformations
ALDH1A2: Aldehyde Dehydrogenase 1 Family Member A2
CYR61: Cysteine-rich angiogenic inducer 61
DGE: Differential gene expression
GFAP: Glial fibrillary acidic protein
ATRA: All retinoic acid
VEGF: Vascular endothelial growth factor
qRT-PCR: Quantitative real-time polymerase chain reaction
RNA: Ribonucleic acid

## References

[1] Achrol AS, Pawlikowska L, McCulloch CE, Poon KY, Ha C, Zaroff JG (2006). Tumor necrosis factor-alpha-238G: a promoter polymorphism is associated with increased risk of new hemorrhage in the natural course of patients with brain arteriovenous malformations. Stroke.

[2] Ajiboye N, Chalouhin N, Starke RM, Zanaty M, Bell R (2014). Cerebral arteriovenous malformations: evaluation and management. Scientific World Journal.

[3] Aucourt J, Jissendi P, Kerdraon O, Baroncini M (2012). Neuroimaging features and pathology of mixed glioblastoma–AVM complex: a case report. J Neuroradiol.

[4] Babic AM, Kireeva ML, Kolesnikova TV, Lau LF (1998). CYR61, a product of a growth factor-inducible immediate early gene, promotes angiogenesis and tumor growth. Proc Natl Acad Sci U S A.

[5] Blebea J, Vu JH, Assadnia S, McLaughlin PJ, Atnip RG, Zagon IS (2002). Differential effects of vascular growth factors on arterial and venous angio-genesis. J VascSurg.

[6] Campos B, Centner FS, Bermejo JL, Ali R, Dorsch K, Wan F (2011). Aberrant expression of retinoic acid signaling molecules influences patient survival in astrocyticgliomas. Am J Pathol.

[7] Chaqour B (2016). Regulating the regulators of angiogenesis by CCN1 and taking it up a Notch. J Cell Commun Signal.

[8] Chen CC, Mo FE, Lau LF (2001). The angiogenic factor Cyr61 activates a genetic program for wound healing in human skin fibroblasts. J Biol Chem.

[9] Chen Y, Zhu W, Bollen AW, Lawton MT, Barbaro NM, Dowd CF (2008). Evidence of inflammatory cell involvement in brain arteriovenous malformations. Neurosurg.

[10] Ellis JA, Mejia Munne JC, Lavine SD, Meyers PM, Connolly ES Jr, Solomon RA. Arteriovenous malformations and headache. J Clin Neurosci. 2016;23:38–43. 10.1016/j.jocn.2015.08.003. Epub 2015 Oct 13. 10.1016/j.jocn.2015.08.003 26461909

[11] Gaetano C, Catalano A, Illi B, Felici A, Minucci S, Palumbo R (2001). Retinoids induce fibroblast growth factor-2 production in endothelial cells via retinoic acid receptor activation and stimulate angiogenesis *invitro* and *in vivo*. Circ Res.

[12] Galou M, Guyon EC, Ensergueix D, Ridet JL (1996). Disrupted glial fibrillary acidic protein network in astrocytes from vimentin knockout mice. J Cell Biol.

[13] Gloviczki P, Duncan A, Kalra M, Oderich G, Ricotta J, Bower T, McKusick M, Bjarnason H, Driscoll D. Vascular malformations: an update. Perspect Vasc Surg Endovasc Ther. 2009;21(2):133–48. 10.1177/1531003509343019. 10.1177/1531003509343019 19713211

[14] Hou F, Dai Y, Suen JY, Fan C, Saad AG, Richter GT (2013). A xenograft animal model of human arteriovenous malformations. Orphanet J Rare Dis.

[15] Isoda K, Fukuda H, Takamura N, Hamamoto Y (1981). Arteriovenous malformation of the brain: histological study and micrometric measurement of abnormal vessels. Acta Pathol Jpn.

[16] Kacem K, Lacombe P, Seylaz J, Bonvento G (1998). Structural organization of the perivascular astrocyte end feet and their relationship with the endothelial glucose transporter: a confocal microscopy study. Glia.

[17] Lai G, Muller KA, Carter BS, Chen CC. Arteriovenous malformation within an isocitrate dehydrogenase 1 mutated anaplastic oligodendroglioma. Surg Neurol Int. 2015;6(Suppl 9):S295-9. 10.4103/2152-7806.159373. 10.4103/2152-7806.159373 PMC4496836 26167373

[18] Lombardi D, Scheithauer BW, Piepgras D, Meyer FB, Forbes GS (1991). “Angioglioma” and the arteriovenous malformation-glioma association. J Neurosurg.

[19] Majewski S, Szmurlo A, Marczak M, Jablonska S, Bollag W (1993). Inhibition of tumor cell-induced angiogenesis by retinoids, 1,25-dihydroxyvitamin D3 and their combination. Cancer Lett.

[20] Miller BA, Bass DI, Chern JJ (2015). De novo AVM formation. Childs Nerv Syst.

[21] Miller BA, Bass DI, Chern JJ (2014). Development of a de novo arteriovenous malformation after severe traumatic brain injury. J Neurosurg Pediatr.

[22] Moftakhar P, Hauptman JS, Malkasian D, Martin NA (2009). Cerebral arteriovenous malformations. Part 1: cellular and molecular biology. Neurosurg Focus.

[23] Mouchtouris N, Jabbour PM, Starke RM, Hasan DM, Zanaty M, Theofanis T (2015). Biology of cerebral arteriovenous malformations with a focus on inflammation. J Cereb Blood Flow Metab.

[24] Naganska E, Matyja E, Pucko E, Zabek M (2013). The coexistence of pleomorphic xanthoastrocytoma and arteriovenous malformation. A case report. Folia Neuropathol.

[25] Pawlikowska L, Tran MN, Achrol AS, McCulloch CE, Ha C, Lind DL (2000). Polymorphisms in genes involved in inflammatory and angiogenic pathways and the risk of hemorrhagic presentation of brain arteriovenous malformations. Stroke.

[26] Saito A, Sugawara A, Uruno A, Kudo M, Kagechika H, Sato Y (2007). All-trans retinoic acid induces in vitro angiogenesis via retinoic acid receptor: possible involvement of paracrine effects of endogenous vascular endothelial growth factor signaling. Endocrinology.

[27] Sasahara A, Kasuya H, Akagawa H, Ujiie H, Kubo O, Sasaki T (2007). Increased expression of ephrin A1 in brain arteriovenous malformation: DNA microarray analysis. Neurosurg Rev.

[28] Schinkel AH (1999). P-glycoprotein, a gatekeeper in the blood-brain barrier. Adv Drug Deliv Rev.

[29] Seker A, Yildirim O, Kurtkaya O, Sav A, Gunel M, Pamir MN, Kiliç T (2006). Expression of integrins in cerebral arteriovenous and cavernous malformations. Neurosurg.

[30] Sirko S, Irmler M, Gascón S, Bek S, Schneider S, Dimou L (2015). Astrocyte reactivity after brain injury-: The role of galectins 1 and 3. Glia.

[31] Sonstein WJ, Kader A, Michelsen WJ, Llena JF, Hirano A, Casper D (1996). Expression of vascular endothelial growth factor in pediatric and adult cerebral arteriovenous malformations: an immunocytochemical study. J Neurosurg.

[32] Thomas JM, Surendran S, Abraham M, Sasankan D, Bhaadri S, Rajavelu A, et al. Gene expression analysis of nidus of cerebral arteriovenous malformations reveals vascular structures with deficient differentiation and maturation. Plos one. 2018;13(6):e0198617. 10.1371/journal.pone.0198617 PMC5999265 29897969

[33] Xie D, Yin D, Tong X, O'Kelly J, Mori A, Miller C (2005). Cyr61 is overexpressed in gliomas and involved in integrin-linked kinase-mediated Akt and beta-catenin-TCF/Lef signaling pathways. Cancer Res.

[34] Zhang C, Harder DR (2002). Cerebral capillary endothelial cell mitogenesis and morphogenesis induced by astrocytic epoxy eicosa trienoic acid. Stroke.

[35] Zhou D, Herrick DJ, Rosenbloom J, Chaqour B (2005). Cyr61 mediates the expression of VEGF, alphav-integrin, and alpha-actin genes through cytoskeletally based mechano transduction mechanisms in bladder smooth muscle cells. J Appl Physiol.

