# Supplementary material for: Aberrant regulation of retinoic acid signaling genes in cerebral arterio venous malformation nidus and neighboring astrocytes
Source: J Neuroinflammation. 2021 Mar 1;18:61. doi: 10.1186/s12974-021-02094-2 (PMC7923665; doi:10.1186/s12974-021-02094-2)
Supplement: Supplementary file 1 — Additional file 1: Supplementary Figure 1. (A) Formaldehyde denature agarose gel depicts the quality and intactness of RNA isolated from control and test (AVM nidus) tissues. (B) The library prepared from fragmented RNA of control sample and the quality of the library was verified using Agilent tap station. (C) The library prepared from fragmented RNA of AVM nidus sample and the quality of the library was verified using Agilent tap station. Supplementary Figure 2. (A) WEGO plot representing the up-regulated genes in AVM tissue compared to control tissue. (B) WEGO plot representing the down-regulated gene in AVM tissue compared to control tissue. Supplementary Figure 3. The KEGG pathway analysis of AVM tissue sample and control tissue sample of RNA sequencing data. Supplementary Figure 4. The immunofluorescence analysis with the secondary antibodies as control for AVM tissues. All the three secondary antibodies used in the analysis has zero background signal. The Hoechst staining depicts the nuclear staining of the intact cells. [file 12974_2021_2094_MOESM1_ESM.docx]

**Supplementary information for**

**Aberrant regulation of retinoic acid signaling genes in cerebral Arterio Venous Malformation nidus and neighbouring astrocytes**

**Jaya Mary Thomas, Dhakshmi Sasankan, Sumi Surendran, Mathew Abraham, Arumugam Rajavelu*, Chandrasekharan C Kartha***

*Correspondence to be addressed: [arajavelu@rgcb.res.in](mailto:arajavelu@rgcb.res.in) & [cckartha@gmail.com](mailto:cckartha@gmail.com)

**Supplementary figure 1: (A)** Formaldehyde denature agarose gel depicts the quality and intactness of RNA isolated from control and test (AVM nidus) tissues. **(B)** The library prepared from fragmented RNA of control sample and the quality of the library was verified using Agilent tap station. **(C)** The library prepared from fragmented RNA of AVM nidus sample and the quality of the library was verified using Agilent tap station.

**Supplementary figure 2: (A)** WEGO plot representing the up-regulated genes in AVM tissue compared to control tissue. **(B)** WEGO plot representing the down-regulated gene in AVM tissue compared to control tissue.

**Supplementary figure 3:** The KEGG pathway analysis of AVM tissue sample and control tissue sample of RNA sequencing data.

**
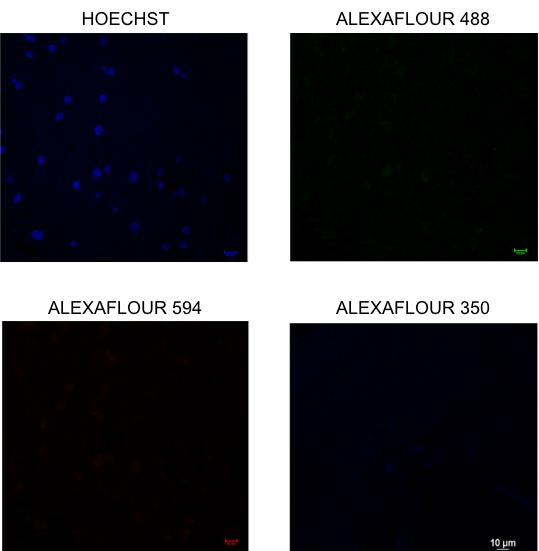
**

**Supplementary figure 4:** The immunofluorescence analysis with the secondary antibodies as control for AVM tissues. All the three secondary antibodies used in the analysis has zero background signal. The Hoechst staining depicts the nuclear staining of the intact cells.

**Supplementary Table 1: List of primers used for qRT-PCR analysis in this study**
