# Supplementary material for: Aberrant regulation of retinoic acid signaling genes in cerebral arterio venous malformation nidus and neighboring astrocytes
Source: J Neuroinflammation. 2021 Mar 1;18:61. doi: 10.1186/s12974-021-02094-2 (PMC7923665; doi:10.1186/s12974-021-02094-2)
Supplement: Supplementary file 2 — Additional file 2: Supplementary Table 1. List of primers used for qRT-PCR analysis in this study. [file 12974_2021_2094_MOESM2_ESM.docx]

**Supplementary Table 1: List of primers used for qRT-PCR analysis in this study**
